# Radiation and Thyroid Cancer

**DOI:** 10.3390/ijms18050911

**Published:** 2017-04-26

**Authors:** Elisabetta Albi, Samuela Cataldi, Andrea Lazzarini, Michela Codini, Tommaso Beccari, Francesco Saverio Ambesi-Impiombato, Francesco Curcio

**Affiliations:** 1 Department of Pharmaceutical Science, University of Perugia, 06123 Perugia, Italy; samuelacataldi@libero.it (S.C.); michela.codini@unipg.it (M.C.); tommaso.beccari@unipg.it (T.B.); 2 Research Center and Analysis Laboratory CRABiON, 06073 Perugia, Italy; andrylazza@gmail.it; 3 Department of Clinical and Biological Sciences, University of Udine, 33100 Udine, Italy; ambesis@me.com (F.S.A.-I.); francesco.curcio@uniud.it (F.C.)

**Keywords:** radiation, thyroid cancer, cancer genes, lipid metabolism

## Abstract

Radiation-induced damage is a complex network of interlinked signaling pathways, which may result in apoptosis, cell cycle arrest, DNA repair, and cancer. The development of thyroid cancer in response to radiation, from nuclear catastrophes to chemotherapy, has long been an object of study. A basic overview of the ionizing and non-ionizing radiation effects of the sensitivity of the thyroid gland on radiation and cancer development has been provided. In this review, we focus our attention on experiments in cell cultures exposed to ionizing radiation, ultraviolet light, and proton beams. Studies on the involvement of specific genes, proteins, and lipids are also reported. This review also describes how lipids are regulated in response to the radiation-induced damage and how they are involved in thyroid cancer etiology, invasion, and migration and how they can be used as both diagnostic markers and drug targets.

## 1. Introduction

Radiation includes ionizing radiation (IR) and non-IR. IR can be distinguished in photon radiation (X- and γ-rays) and particle radiation (such as electrons, protons, neutrons, carbon ions, and alpha and beta particles). IR has enough energy to free electrons from atoms or molecules ionizing them. Non-IR includes ultraviolet (UV), visible light laser, infrared, microwaves, and radio waves.

It is generally accepted that high acute doses of IR may be harmful to living organisms. In radiation accidents, the determination of the radiation dose is a key step for medical decisions and patient prognosis. The estimation of the absorbed dose aids in establishing the risk for acute or chronic health effects, up to months or years after irradiation [1]. The acute radiation syndrome is caused by the exposure to high IR during a short period of time, causing depletion of parenchymal cells in a tissue [2]. Therefore, the doses and duration of radiation exposure are critical for humans. Until now, radiation is largely used in clinical diagnostics and therapy with remarkable clinical benefits for patients. Radiotherapy is essentially based on both X- and γ-rays, which provide photons that are able to specifically penetrate the target and that can be captured on film [3]. Proton therapy uses proton beams that do not traverse the target but stop at an energy-dependent depth within the target with no exit dose [3]. Despite positive diagnostic and therapeutic aspects, the inappropriate use of computed tomography, leading to cancer risk, has been drawing attention for many years [4]. Additionally, the chronic radiation syndrome, ranging from dose-limiting toxicity to the increased risk of secondary cancers following radiation in patients, should always be considered [5]. To this end, adaptive responses to low radiation doses have been widely studied both in vitro and in vivo to ascertain the biological mechanism of radiation action. Radiation-induced signaling pathways in different tissues via EGFR, IGFI-R, PI3K, MAPK, JNK, and p38, as well as via FAS-R, TNF-R, and NFKB, have been reviewed [6].

## 2. Sensitivity of the Thyroid Gland to Radiation and Cancer Development

Although classically considered resistant to acute effects of radiation [7], the thyroid has actually proved to be particularly sensitive to the long-term effects of radiation exposure as demonstrated in studies of human subjects exposed to sublethal radiation doses [8]. More epidemiological studies were performed. It has been demonstrated by analyzing young adults exposed to radiation during childhood. A screening study of 11,970 residents of Belarus aged ≤18 years at the time of the Chernobyl nuclear accident showed a risk for neoplastic nodules significantly higher than for non-neoplastic nodules [9]. High amounts of radiation caused a significant increase in the incidence of thyroid gland carcinoma, as observed in several nuclear catastrophes such as Hiroshima, Nagasaki, Chernobyl, and more recently, Fukushima [10]. The effects of radiation in inducing thyroid nodules have been demonstrated in atomic bomb survivors from 62 to 66 years after exposure during their childhood [11]. The analysis of thyroid consequences of the 2011 Fukushima nuclear reactor accident showed that 35% of the residents developed thyroid nodules and/or cysts [12]. The study of the survivors in Hiroshima and Nagasaki has demonstrated that the risk for thyroid cancer was significantly higher if IR exposure occurred at pediatric ages [9]. Exposure to low or moderate doses of IR appeared to specifically increase the risk of thyroid papillary microcarcinoma, even when exposure occurred during adulthood [13]. Richardson [14] stated that exposure to IR in adulthood was positively associated with thyroid cancer among female survivors from atomic bombs (excess relative rate/Gray (Gy) = 0.70; 90% confidence interval = 0.20–1.46), although the risk seemed to be lower if they were exposed to radiation in their childhood. Ron et al. [15] compared atomic bomb survivors, children treated for tinea capitis, children irradiated for enlarged tonsils, and infants irradiated for an enlarged thymus gland with two case controls of untreated patients with cervical cancer and childhood cancer. The authors reported that the risk to develop thyroid cancer was correlated with age and sex. In fact, in childhood, the pooled excess relative risk per Gy (ERR/Gy) was 7.7 (95% CI = 2.1, 28.7) and the excess absolute risk per 10(4) PY Gy (EAR/10(4) PY Gy) was 4.4 (95% CI = 1.9, 10.1); the excess relative risk was greater (*p* = 0.07) for females than males. Holm affirmed that, usually, the excess relative risk for thyroid cancer started 5–10 years after radiation exposure and continued until at least 40 years after exposure; it was correlated more to the early age (prior to five years of age) than to the sex [16]. Exposure to ^131^I during childhood was associated with an increased risk of thyroid cancer and both iodine deficiency and iodine supplementation appeared to modify such risk [17]. Robbins and Schneider confirmed the importance of the age, youth being a risk factor. Although the clinical use of radioiodine has not been reported to cause thyroid cancer, a low number of patients with cancer were young children and the studied cohorts were too small (consisting of 17 to 191 patients) to provide the statistical power to detect such a relatively rare event [18]. Among 585 patients with neck radiation, seven survivors developed papillary thyroid carcinoma (PTC). This indicates that, in adult survivors of cancer during their childhood or young adulthood with a history of radiation therapy to the neck for cancer, an annual physical exam should be considered appropriate as a thyroid cancer screening strategy [19]. Patients with head and neck squamous cell carcinoma showed a strong incidence of a subsequent primary thyroid cancer during the first 5 years after diagnosis and IR-treatment, supporting the concept that continued surveillance of thyroid status is important in this scenario [20]. Molecular mechanisms (genes, proteins, and lipids) that played a role in radiation-induced damages were reported in the following paragraphs.

## 3. Genes and Proteins Involved in Radiation-Induced Cancer

**Figure 1:**
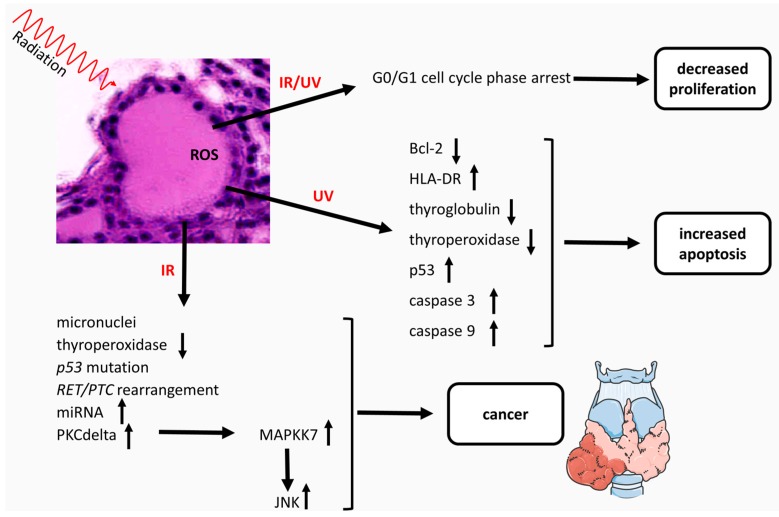
Effects of IR and UV in the thyroid gland. Synopsis of the main literature in the field [22,23,24,25,26,27,28,29,30,31,32,33,34,35,36,37,38,39,40,41,42,43,44,45]. HLA-DR: human leukocyte antigen-DR; *RET/PTC*: rearranged during transfection/papillary thyroid carcinoma; PKC: protein kinase C; MAPKK7: mitogen-activated protein kinase 7; JNK: c-Jun NH2-terminal kinases; IR: ionizing radiation; UV: ultraviolet rays; ROS: reactive oxygen species. Some graphical elements were taken from the Servier Medical Art Library, available from http://www.servier.com/Powerpoint-image-bank under Creative Commons Attribution 3.0 Unported License. Up-arrows, increase; down-arrows, decrease.

Advances in biochemistry and molecular biology have allowed the identification of the IR and non-IR molecular events in the thyroid gland (Figure 1).

Both IR and UV induced enhanced production of free oxygen radicals and modified pro-oxidant states [21]. However, the greatest damage to proteins and nucleic acids were with IR.

### 3.1. Ionizing Radiations

IR directly and/or indirectly causes oxidative stresses to the biological systems at the local or systematic level by influencing aging, genetic destabilization and mutagenicity, membrane lysis and cell death, alteration of enzymatic activities and metabolic events, mitochondrial dysfunction, and cancer [22]. The effects of IR in the thyroid gland have been extensively studied. The chronic exposure of mature rats to low-intensity γ-rays between 5 and 50 cGy (dose rates: 25, 400 µGy/h) induced the formation of micronuclei three times higher in irradiated thyrocytes than in thyrocytes of control animals [23]. Furthermore, the residual thyroid of hemi-thyroidectomized rats exposed to acute γ-rays with 2–4 Gy presented micronuclei. Ermakova et al. found that the presence of micronuclei was also a sensitive indicator of radiation-induced genetic damages in the follicular epithelium of thyroid gland [23]. Moreover, IR delayed follicular thyroid cell proliferation [24]. Thyroids of old rats irradiated in the neck region with an X-ray single dose of 3 Gy showed an increase in proliferating follicular cells two days after irradiation, followed by a phase of sharp decrease in cell proliferation between the 2nd and 6th day after irradiation. During the cell proliferation phase, the cell cycle was shortened by approximately 50%, predominantly due to a decrease of the G1-phase duration [24]. ^131^I was shown to trigger apoptosis in human thyrocytes [25]. The cell viability of human thyroid epithelial cells purified from surgical tissue specimens was not affected by single doses of 5 or 50 Gy IR, and there was no induction of Heat shock proteins (HSP)-72, as an indicator of acute cellular stress. Nevertheless, the expression of thyroperoxidase (TPO), a key enzyme of thyroid hormone synthesis, significantly decreased [26]. The authors hypothesized that the suppression of thyroid hormone synthesis due to TPO reduction could contribute to an early development of thyroid dysfunction following irradiation, and they recommended considering this effect during radiation therapy [26]. On the other hand, the thyroid hormone modulation with X-rays induced neoplastic transformation in vitro [27]. Mizuno et al. [28] indicated that IR caused various oncogene activations, with specificity for early gene alteration uniquely associated with thyroid carcinogenesis. Irradiation of a non-tumorigenic human thyroid epithelial cell line with α-particles or γ-rays stimulated Exons 6 and 7, as well as *p53* mutations in the childhood PTC in Belarus, presumably as a result of radioiodine fallout [29]. In addition, IR exposure of cultured human thyroid cells stimulated the induction of c-Jun NH_2_-terminal kinases (JNK) activity, not extracellular signal-regulated kinases (ERK) activity, to a 3.5-fold extent. The effect was specific for thyroid cells as it was absent in fibroblasts [30]. The JNK activation was mediated at least partially through a protein kinase C (PKC)-dependent pathway [30]. Mitsutake et al. [31] reported that among PKC-α, β2, δ, ε, and ζ isoforms expressed in primary cultured human thyroid cells, only PKC-δ was involved in an IR-induced JNK activation. Moreover, PKC-δ acted via mitogen-activated protein kinase kinase 7 (MAPKK7), not via MAPKK4 [31]. Characteristically, IR was responsible for a dose-dependent REarranged during Transfection/Papillary Thyroid Cance (*RET/PTC*) rearrangement in human thyroid cells [32]. Ameziane et al. [33] demonstrated that this effect was dependent on generated H_2_O_2_ during irradiation; it was responsible for the breaks of double-strand DNA and facilitated *RET/PTC*1 formation. As a consequence, by pretreating the cells with catalase, a scavenger of H_2_O_2_, *RET/PTC*1 rearrangement was decreased. Cells derived from the neural crest, kidney, and enteric nervous system expressed *RET* proto-oncogene [34]. Hamatani et al. [35] reported that in PTC the *RET* proto-oncogene generated a series of chimeric-transforming oncogenes collectively described as *RET/PTCs*. In childhood PTC with a history of radiation exposure, *RET/PTC* rearrangements represented a major event and among atomic bomb survivors, the frequency of rearrangements increased in relation to an increase of radiation dose [35]. In two studies that employed human fetal thyroid tissue xenografts, Mizuno et al. [28] demonstrated that X-ray irradiation generated both *RET/PTC*1 and *RET/PTC*3 rearrangements, and the *RET/PTC*1 type was the most common. Notably, patients exposed to Chernobyl radiation developed PTC, and survivors of the atomic bomb in Japan had a very high frequency of *RET/PTC* chromosomal rearrangement [36]. In addiction, B-Raf proto-oncogene (BRAF) mutation (BRAF V600E) was associated with PTC [37]. Guan et al. [38] demonstrated that high iodine intake was a significant risk factor for BRAF V600E mutation and the development of PTC in the thyroid gland. The prevalence of BRAF V600E mutation in pediatric PTC was significantly lower than that in adults, 54% versus 85% [39]. On the other hand, a clinicopathological study showed that BRAF V600E was associated with older age and larger tumor size [40]. In patients with PTC who were 0–18 years at the time of the Chernobyl accident, BRAF V600E mutation was present, but the percentage was less than that of *RET/PTC*1 and *RET/PTC*3 rearrangements t [41]. Genomic copy number alterations of PTC of the Ukrainian-American cohort after the Chernobyl accident were associated with BRAF V600E mutation [42]. BRAFV600E mutation was less frequent in the cases of Hiroshima and Nagasaki survivors exposed to higher radiation doses [35]. In atomic bomb survivors in Hiroshima, the median radiation dose able to induce PTC was significantly lower in patients with BRAFV600E mutation than that without the mutation [43]. A screening program of various genetic alterations in children aged 0–18 years old at the time of the Fukushima accident showed that BRAF V600E was highly prevalent in the 63.2% of the population [44]. The difference of the data in various atomic bomb survivors could be due to the different genetic profile of patients, considering that the response to radiation of the thyroid gland was dependent on the genetic profile of the patients [44]. For this reason, Fukushima PTC was completely different from post-Chernobyl radiation-induced PTC [44], indicating the possibility of non-radiogenic etiology of PTC. Significant upregulation of a subset of these miRNAs (miR-187, miR-146b, and miR-155) was found to be more pronounced in PTC carrying *RET/PTC* rearrangements [45]. The association between miRNAs and radiation exposure has been reported in a variety of mouse tissues, including spleen, colon, thymus, and kidney [46]. Acute exposure of thyroid cells to γ-radiation resulted in several specific patterns of miRNA response not directly associated to carcinogenesis [47].

### 3.2. Non-Ionizing Radiations

UV radiation induced apoptosis in the FRTL-5 rat thyroid cell line [48] by the increase of p53 and caspases 3 and 9, and the decrease of Bcl-2, together with a transient but significant increase in HLA-DR expression [49] and the impairment of genes involved in thyroid hormone production, such as genes for thyroglobulin and TPO [50]. The effect was dependent on TSH that stimulates cell proliferation. Overall, TSH starvation induced virtually all cells to accumulate in the G0/G1 cell cycle phase, blocking cell proliferation, and rendering cells more resistant to UV-C radiation-induced apoptosis [51]. Thus, the effect of UV on FRTL-5 cells in culture was strongly related to the physiological state of the cells. Proliferating cells were more sensitive to radiation treatment than quiescent cells; the cells in a proapoptotic state caused by the lack of trophic support were less sensitive to radiation treatment [52].

## 4. Lipids as Regulators of Radiation-Induced Cancer

**Figure 2:**
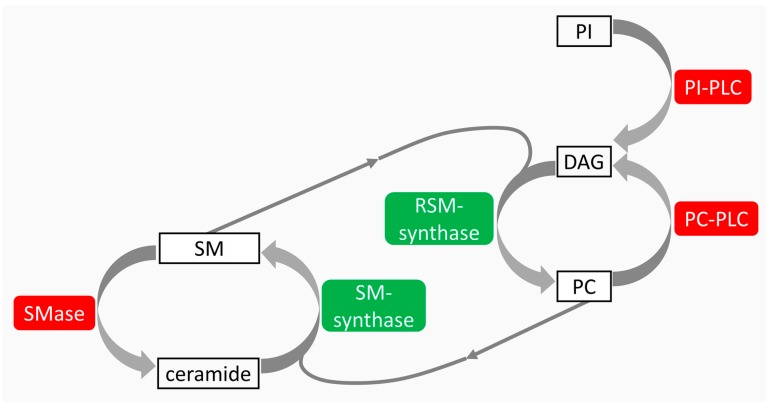
Cross talk among sphingomyelin (SM), phospatidylcholine (PC), and phosphatidylinositol (PI) metabolism. SM is degraded by sphingomyelinase (SMase) to produce ceramide and is restored by sphingomyelin-synthase (SM-synthase) from PC and ceramide. PC is degraded by phosphatidylcholine-specific phospholipase C (PC-PLC) to produce diacylglycerol (DAG) and is restored by reverse sphingomyelin-synthase (RSM-synthase) from SM and DAG. PI is degraded by phosphatidylinositol-specific phospholipase C (PI-PLC). In red, catabolic enzymes; in green, anabolic enzymes; thin arrows, SM for PC synthesis and PC for SM synthesis; thick arrows, the relation of PI and PC with DAG and the relation of SM with ceramide.

Differences in the responses to IR and non-IR of proliferating, quiescent and proapoptotic thyroid cells were associated with a very complex mechanism of lipid metabolism. A specific cross-talk exists among sphingomyelin (SM), phosphatidylcholine (PC), and phosphatidylinositol (PI) in both cell membrane and nuclei [53,54] (Figure 2).

### 4.1. Ionizing Radiation

After IR-exposure, ceramide and diacylglycerol (DAG) acted as second messengers inducing proapoptotic and antiapoptotic signals, respectively [55]. FRTL-5 cells submitted to accelerated proton beams (CERN, Geneva, Switzerland) showed changes of lipid metabolism enzymes [55]. Proton beams induced quiescent thyroid cells towards a proapoptotic state and proliferating thyroid cells towards an initial apoptotic state, by altering the nuclear SM-metabolism. In cell nuclei the strong activation of neutral-sphingomyelinase (N-SMase) reduced SM content that was important for the DNA-stability. The ceramide produced in the nucleus probably was translocated to the cytoplasm, where it could be metabolized to sphingosine and sphingosine-1-phosphate, lipid mediators involved in apoptosis [56].

### 4.2. Non-Ionizing Radiation

UV radiation enriched the ceramide pool due to acid-sphingomyelinase (A-SMase) and N-SMase activities and enlarged the DAG pool due to phosphatidylcholine-specific phospholipase C (PC-PLC) and phosphatidylinositol-specific phospholipase C (PI-PLC) in cell membranes of proliferating cells [52]. In purified nuclei, radiation stimulated N-SMase and reverse SM-synthase (RSM-synthase) activities while inhibited PC-PLC, PI-PLC, and SM-synthase activities leading to further ceramide pool enrichment and DAG pool reduction. The effect of UV irradiation on lipid metabolism was higher in the nucleus than in cell membranes [52]. The effect on nuclear lipids was very relevant, because of their role in cell proliferation, differentiation, and apoptosis [57] by acting as a platform for the attachment [58,59] and regulation [60] of active chromatin and for nuclear drug activity [61,62]. The prolonged presence in the stratosphere results in exposure to radiation, so stratospheric balloons were used to expose FRTL-5 cells to radiation present at a 30–40 km altitude for approximately 20 h (BIRBA mission). In proliferating cells, low doses of stratospheric radiation did not induce cell death but only early modifications of nuclear SM and PC metabolism. In purified nuclei, SMase and RSM-synthase activities were increased, whereas PC-PLC and SM-synthase activities were inhibited, leading to an increase of the ceramide/DAG ratio [63]. These studies indicated that nuclear SM metabolism was involved in radiation-induced damage (Table 1). The results were relevant considering the possibility that radiation induced thyroid cancer.

## 5. Biomarkers of Thyroid Damage

Considering the molecular effects of radiation on the thyroid gland, the analysis of micronuclei frequency in peripheral blood lymphocytes is applicable as a biomarker of chromosomal damage, genome instability, and cancer risk [64]. A negative correlation between micronuclei frequency and the level of platelets without correlation to thyroid-related hormones has been observed in blood of patients suffering from differentiated thyroid cancer and treated with radioactive iodine (^131^I) [65]. Dom et al. [66] studied children exposed and non-exposed to the Chernobyl radiation and compared them in the transcriptomes of normal contralateral tissues of PTC; in this way, the authors identified a gene expression signature (793 probes) that permits discrimination between both cohorts. To differentiate radiation and no radiation-induced PTC, Port et al. investigated the RNA isolated from 11 post-Chernobyl PTCs and 41 sporadic PTCs [67]. The microarray detected 646 upregulated genes and 677 downregulated genes [67]. The analysis of gene expression can be useful to measure the predisposition to developing cancer after radiation exposure [68]. In particular, the overexpression of the CLIP2 gene is the most promising marker; in fact, it was found in the majority of PTCs from young patients included in the Chernobyl tissue bank [69]. In post-Chernobyl PTC, the expression of CLIP2 gene was radiation dose-dependent [70]. The use of CLIP2 as radiation biomarker was supported by a study indicating its involvement in the fundamental carcinogenic processes including apoptosis, mitogen-activated protein kinase signaling, and genomic instability [71]. In comparison with normal tissues, thyroid carcinoma tissues from patients had a significant increase in lecithin, SM, and cholesterol [72]. Changes of the SM content together with other lipids in the blood plasma of patients with thyroid carcinoma were reported [73]. Serum lipidomic profiling with a panel which included 36:3phosphatidic acid (PA) and 34:1SM can be useful to distinguish between malignant thyroid cancer and benign thyroid tumors [74]. Rath et al. found that glycosylation of ceramide could contribute to the drug-resistance phenotype in thyroid malignancies [75]. Furthermore, it has been suggested that sphingosine kinase 1 (SphK1) and sphingosine-1-phosphate (S1P) may be relevant in the etiology of thyroid cancer, and in the regulation of both invasion and migration of thyroid cancer cells. Therefore, their contents could be useful as specific biomarkers of cancer transformation and progression [76].

## 6. Conclusions

To date, a number of specific molecular targets have been identified, by which radiation exerts its effects upon the thyroid gland, inducing long-term damages including cancer. Many genes, proteins, and lipids are involved in the mechanism of action, effects, and consequences of radiation, so this field of study is still widely open. It is becoming increasingly evident in the most recent literature that specific genes, proteins, and lipids are important targets of both radiation and cancer, but many points remain obscure. Further studies are required to shed more light on the complexity of interactions among various cellular components.

## Figures and Tables

**Table 1:** The effect of radiation types on nuclear lipid metabolism.

FRTL-5 Nuclei				
Radiation	Proliferating Cells	Quiescent Cells	Proapoptotic Cells	References
UV	↑ SMase ++	↑ SMase +	↑ SMase ++	[46]
	↑ RSMase ++	↑ RSMase ++	↑ RSMase ++	
	↓ SMsynthase ++	↓ SMsynthase +	↓ SMsynthase ++	
	↓ PCPLC ++	↓ PCPLC +	↓ PCPLC ++	
	↓ PIPLC ++	↓ PIPLC +	↓ PIPLC ++	
Stratosphere	↑ SMase ++	↑ SMase +		[57]
	↑ RSMase ++	↑ RSMase ++		
	↓ SMsynthase ++	↓ SMsynthase ++		
	↓ PCPLC ++	↓ PCPLC +		
Protons	↑ SMase +++	↑ SMase =		[50]
	↓ SMsynthase =	↓ SMsynthase ++		

SMase: sphingomyelinase; RSMase: reverse sphingomyelin-synthase; SMsynthase: sphingomyelin-synthase; PCPLC: phosphatidylcholine-specific phospholipase C; PIPLC: phosphatidylinositol-specific phospholipase C; + low change; ++ medium change; +++ high change. ↑ increased activity; ↓ decreased activity. =: In protons proliferating cell SMsynthase ↓ should be deleted; in protons quiescent cells SMase ↑ should be deleted.

## Abbreviations

A-SMase	Acid-sphingomyelinase
DAG	Diacylglycerol
HLA-DR	Human leukocyte antigen-DR
IR	Ionizing radiation
JNK	c-Jun NH2-terminal kinases
MAPKK7	Mitogen-activated protein kinase kinase 7
N-SMase	Neutral-sphingomyelinase
PC	Phosphatidylcholine
PC-PLC	Phosphatidylcholine-specific phospholipase C
PI	Phosphatidylinositol
PI-PLC	Phosphatidylinositol-specific phospholipase C
PKC	Protein kinase C
PTC	Papillary thyroid carcinoma
RET	Rearranged during transfection
RSM-synthase	Reverse sphingomyelin-synthase
SM	Sphingomyelin
SM-synthase	Sphingomyelin-synthase
UV	Ultraviolet rays

## References

[1] Port M., Herodin F., Valente M., Drouet M., Ullmann R., Doucha-Senf S., Lamkowski A., Majewski M., Abend M. (2016). MicroRNA expression for early prediction of late occurring hematologic acute radiation syndrome in baboons. PLoS ONE.

[2] Kazzi Z., Buzzell J., Bertelli L., Christensen D. (2015). Emergency department management of patients internally contaminated with radioactive material. Emerg. Med. Clin. N. Am..

[3] Yamoah K., Johnstone P.A. (2016). Proton beam therapy: Clinical utility and current status in prostate cancer. OncoTargets Ther..

[4] Frush D. (2016). MO-FG-207A-03: Radiation and cancer perspectives from the trenches: Are we providing care or promoting scare?. Med. Phys..

[5] Orton C., Borras C., Carlson D. (2014). Radiation biology for radiation therapy physicists. Med. Phys..

[6] Dent P., Yacoub A., Contessa J., Caron R., Amorino G., Valerie K., Hagan M.P., Grant S., Schmidt-Ullrich R. (2003). Stress and radiation-induced activation of multiple intracellular signaling pathways. Radiat. Res..

[7] Rubin P., Casarett G.W. (1968). Clinical radiation pathology as applied to curative radiotherapy. Cancer.

[8] Paro J.N., Zavisić B.K. (2012). Iodine and thyroid gland with or without nuclear catastrophe. Med. Pregl..

[9] Cahoon E.K., Nadirov E.A., Polanskaya O.N., Yauseyenka V.V., Velalkin I.V., Yeudachkova T.I., Maskvicheva T.I., Minenko V.F., Liu W., Drozdovitch V. (2017). Risk of thyroid nodules in residents of Belarus exposed to Chernobyl fallout as children and adolescents. J. Clin. Endocrinol. Metab..

[10] Imaizumi M., Ohishi W., Nakashima E., Sera N., Neriishi K., Yamada M., Tatsukawa Y., Takahashi I., Fujiwara S., Sugino K.T. (2015). Association of radiation dose with prevalence of thyroid nodules among atomic bomb survivors exposed in childhood (2007–2011). JAMA Intern. Med..

[11] Nagataki S. (2012). Thyroid consequences of the Fukushima nuclear reactor accident. Eur. Thyroid J..

[12] Furukawa K., Preston D., Funamoto S., Yonehara S., Ito M., Tokuoka S., Sugiyama H., Soda M., Ozasa K., Mabuchi K. (2013). Long-term trend of thyroid cancer risk among Japanese atomic-bomb survivors: 60 Years after exposure. Int. J. Cancer.

[13] Hayashi Y., Lagarde F., Tsuda N., Funamoto S., Preston D.L., Koyama K., Mabuchi K., Ron E., Kodama K., Tokuoka S. (2010). Papillary microcarcinoma of the thyroid among atomic bomb survivors: Tumor characteristics and radiation risk. Cancer.

[14] Richardson D.B. (2009). Exposure to ionizing radiation in adulthood and thyroid cancer incidence. Epidemiology.

[15] Ron E., Lubin J.H., Shore R.E., Mabuchi K., Modan B., Pottern L.M., Schneider A.B., Tucker M.A., Boice J.D. (1995). Thyroid cancer after exposure to external radiation: A pooled analysis of seven studies. J. Radiat. Res..

[16] Holm L.E. (1991). Radiation-induced thyroid neoplasia. Sozial und Präventivmedizin.

[17] Cardis E., Kesminiene A., Ivanov V., Malakhova I., Shibata Y., Khrouch V., Drozdovitch V., Maceika E., Zvonova I., Vlassov O. (2005). Risk of thyroid cancer after exposure to 131I in childhood. J. Natl. Cancer Inst..

[18] Robbins J., Schneider A.B. (2000). Thyroid cancer following exposure to radioactive iodine. Rev. Endocr. Metab. Disord..

[19] Tonorezos E.S., Barnea D., Moskowitz C.S., Chou J.F., Sklar C.A., Elkin E.B., Wong R.J., Li D., Tuttle R.M., Korenstein D. (2016). Screening for thyroid cancer in survivors of childhood and young adult cancer treated with neck radiation. J. Cancer Surviv..

[20] Chan J.Y., Gooi Z., Mydlarz W.K., Agrawal N. (2016). Risk of thyroid malignancy following an index head and neck squamous cell carcinoma: A population-based study. Ear Nose Throat J..

[21] Borek C. (1993). Molecular mechanisms in cancer induction and prevention. Environ. Health Perspect..

[22] Islam M.T. (2017). Radiation interactions with biological system. Int. J. Radiat. Biol..

[23] Ermakova O.V., Pavlov A.V., Korableva T.V. (2008). Cytogenetic effects in follicular epithelium of thyroid gland under prolonged exposure to gamma-radiation at low-doses. Radiat. Biol. Radioecol..

[24] Christov K. (1982). Effect of irradiation on the proliferation kinetics of thyroid follicular cells in infant rats. Exp. Pathol..

[25] Russo E., Guerra A., Marotta V., Faggiano A., Colao A., del Vecchio S., Tonacchera M., Vitale M. (2013). Radioiodide induces apoptosis in human thyroid tissue in culture. Endocrine.

[26] Blasko I., Sztankay A., Lukas P., Grubeck-Loebenstein B. (2000). Decreased thyroid peroxidase expression in cultured thyrocytes after external gamma irradiation. Exp. Clin. Endocrinol. Diabetes.

[27] Guernsey D.L., Ong A., Borek C. (1980). Thyroid hormone modulation of X ray-induced in vitro neoplastic transformation. Nature.

[28] Mizuno T., Kyoizumi S., Suzuki T., Iwamoto K.S., Seyama T. (1997). Continued expression of a tissue specific activated oncogene in the early steps of radiation-induced human thyroid carcinogenesis. Oncogene.

[29] Gamble S.C., Cook M.C., Riches A.C., Herceg Z., Bryant P.E., Arrand J.E. (1999). p53 mutations in tumors derived from irradiated human thyroid epithelial cells. Mutat. Res..

[30] Hara T., Namba H., Yang T.T., Nagayama Y., Fukata S., Kuma K., Ishikawa N., Ito K., Yamashita S. (1998). Ionizing radiation activates c-Jun NH_2_-terminal kinase (JNK/SAPK) via a PKC-dependent pathway in human thyroid cells. Biochem. Biophys. Res. Commun..

[31] Mitsutake N., Namba H., Shklyaev S.S., Tsukazaki T., Ohtsuru A., Ohba M., Kuroki T., Ayabe H., Yamashita S. (2001). PKC delta mediates ionizing radiation-induced activation of c-Jun NH_2_-terminal kinase through MKK7 in human thyroid cells. Oncogene.

[32] Caudill C.M., Zhu Z., Ciampi R., Stringer J.R., Nikiforov Y.E. (2005). Dose-dependent generation of RET/papillary thyroid carcinoma in human thyroid cells after in vitro exposure to γ-radiation: A model of carcinogenic chromosomal rearrangement induced by ionizing radiation. J. Clin. Endocrinol. Metab..

[33] Ameziane-El-Hassani R., Boufraqech M., Lagente-Chevallier O., Weyemi U., Talbot M., Métivier D., Courtin F., Bidart J.M., El Mzibri M., Schlumberger M. (2010). Role of H_2_O_2_ in *RET/PTC*1 chromosomal rearrangement produced by ionizing radiation in human thyroid cells. Cancer Res..

[34] Schuchardt A., D’Agati V., Larsson-Blomberg L., Costantini F., Pachnis V. (1994). Defects in the kidney and enteric nervous system of mice lacking the tyrosine kinase receptor Ret. Nature.

[35] Hamatani K., Eguchi H., Ito R., Mukai M., Takahashi K., Taga M., Imai K., Cologne J., Soda M., Arihiro K. (2008). *RET/PTC* rearrangements preferentially occurred in papillary thyroid cancer among atomic bomb survivors exposed to high radiation dose. Cancer Res..

[36] Mizuno T., Iwamoto K.S., Kyoizumi S., Nagamura H., Shinohara T., Koyama K., Seyama T., Hamatani K. (2000). Preferential induction of *RET/PTC*1 rearrangement by X-ray irradiation. Oncogene.

[37] Daliri M., Abbaszadegan M.R., Bahar M.M., Arabi A., Yadollahi M., Ghafari A., Taghehchian N., Zakavi S.R. (2014). The role of BRAF V600E mutation as a potential marker for prognostic stratification of papillary thyroid carcinoma: A long-term follow-up study. Endocr. Res..

[38] Guan H., Ji M., Bao R., Yu H., Wang Y., Hou P., Zhang Y., Shan Z., Teng W., Xing M. (2009). Association of high iodine intake with the T1799A BRAF mutation in papillary thyroid cancer. J. Clin. Endocrinol. Metab..

[39] Oishi N., Kondo T., Nakazawa T., Mochizuki K., Inoue T., Kasai K., Tahara I., Yabuta T., Hirokawa M., Miyauchi A. (2017). Frequent BRAF V600E and absence of TERT promoter mutations characterize sporadic pediatric papillary thyroid carcinomas in Japan. Endocr. Pathol..

[40] Cordioli M.I., Moraes L., Bastos A.U., Besson P., Alves M.T., Delcelo R., Monte O., Longui C., Cury A.N., Cerutti J.M. (2017). Fusion Oncogenes are the main genetic events found in sporadic papillary thyroid carcinomas from children. Thyroid.

[41] Tronko M., Bogdanova T., Voskoboynyk L., Zurnadzhy L., Shpak V., Gulak L. (2010). Radiation induced thyroid cancer: Fundamental and applied aspects. Exp. Oncol..

[42] Selmansberger M., Braselmann H., Hess J., Bogdanova T., Abend M., Tronko M., Brenner A., Zitzelsberger H., Unger K. (2015). Genomic copy number analysis of Chernobyl papillary thyroid carcinoma in the Ukrainian-American Cohort. Carcinogenesis.

[43] Takahashi K., Eguchi H., Arihiro K., Ito R., Koyama K., Soda M., Cologne J., Hayashi Y., Nakata Y., Nakachi K. (2007). The presence of BRAF point mutation in adult papillary thyroid carcinomas from atomic bomb survivors correlates with radiation dose. Mol. Carcinog..

[44] Mitsutake N., Fukushima T., Matsuse M., Rogounovitch T., Saenko V., Uchino S., Ito M., Suzuki K., Suzuki S., Yamashita S. (2015). BRAF^V600E^ mutation is highly prevalent in thyroid carcinomas in the young population in Fukushima: A different oncogenic profile from Chernobyl. Sci. Rep..

[45] Nikiforova M.N., Tseng G.C., Steward D., Diorio D., Nikiforov Y.E. (2008). MicroRNA expression profiling of thyroid tumors: Biological significance and diagnostic utility. J. Clin. Endocrinol. Metab..

[46] He L., He X., Lim L.P., de Stanchina E., Xuan Z., Liang Y., Xue W., Zender L., Magnus J., Ridzon D. (2007). A microRNA component of the p53 tumour suppressor network. Nature.

[47] Nikiforova M.N., Gandhi M., Kelly L., Nikiforov Y.E. (2011). MicroRNA dysregulation in human thyroidcells following exposure to ionizing radiation. Thyroid.

[48] Del Terra E., Francesconi A., Meli A., Ambesi-Impiombato F.S. (2001). Radiation-dependent apoptosis on cultured thyroid cells. Phys. Med..

[49] Kostic I., Toffoletto B., Toller M., Beltrami C.A., Ambesi-Impiombato F.S., Curcio F. (2010). UVC radiation-induced effect on human primary thyroid cell proliferation and HLA-DR expression. Horm. Metab. Res..

[50] Baldini E., D’Armiento M., Sorrenti S., del Sordo M., Mocini R., Morrone S., Gnessi L., Curcio F., Ulisse S. (2013). Effects of ultravioletradiation on FRTL-5 cell growth and thyroid-specific gene expression. Astrobiology.

[51] Del Terra E., Francesconi A., Donnini D., Curcio F., Ambesi-Impiombato F.S. (2003). Thyrotropin effects on ultravioletradiation-dependent apoptosis in FRTL-5 cells. Thyroid.

[52] Albi E., Cataldi S., Rossi G., Viola Magni M., Toller M., Casani S., Perrella G. (2008). The nuclear ceramide/diacylglycerol balance depends on the physiological state of thyroidcells and changes during UV-C radiation-induced apoptosis. Arch. Biochem. Biophys..

[53] Albi E., Lazzarini R., Viola Magni M. (2008). Phosphatidylcholine/sphingomyelinmetabolismcrosstalk inside the nucleus. Biochem. J..

[54] Albi E., Rossi G., Maraldi N.M., Viola Magni M., Cataldi S., Solimando L., Zini N. (2003). Involvement of nuclear phosphatidylinositol-dependent phospholipases C in cell cycle progression during rat liver regeneration. J. Cell. Physiol..

[55] Sautin Y., Takamura N., Shklyaev S., Nagayama Y., Ohtsuru A., Namba H., Yamashita S. (2000). Ceramide-induced apoptosis of human thyroid cancer cells resistant to apoptosis by irradiation. Thyroid.

[56] Albi E., Perrella G., Lazzarini A., Cataldi S., Lazzarini R., Floridi A., Ambesi-Impiombato F. S., Curcio F. (2014). Critical role for the protons in FRTL-5 thyroid cells: Nuclear sphingomyelinase induced-damage. Int. J. Mol. Sci..

[57] Albi E., Viola Magni M.P. (2004). The role of intranuclear lipids. Biol. Cell.

[58] Cascianelli G., Villani M., Tosti M., Marini F., Bartoccini E., Magni M.V., Albi E. (2008). Lipid microdomains in cell nucleus. Mol. Biol. Cell.

[59] Albi E., Villani M. (2009). Nuclear lipid microdomains regulate cell function. Commun. Integr. Biol..

[60] Albi E., Lazzarini A., Lazzarini R., Floridi A., Damaskopoulou E., Curcio F., Cataldi S. (2013). Nuclear lipid microdomain as place of interaction between sphingomyelin and DNA during liver regeneration. Int. J. Mol. Sci..

[61] Bartoccini E., Marini F., Damaskopoulou E., Lazzarini R., Cataldi S., Cascianelli G., Gil Garcia M., Albi E. (2011). Nuclear lipid microdomain sregulate nuclear vitamin D3 uptake and influence embryonic hippocampal cell differentiation. Mol. Biol. Cell.

[62] Cataldi S., Codini M., Cascianelli G., Tringali S., Tringali A.R., Lazzarini A., Floridi A., Bartoccini E., Garcia-Gil M., Lazzarini R. (2014). Nuclear lipid microdomain as resting place of dexamethasone to impair cell proliferation. Int. J. Mol. Sci..

[63] Albi E., Cataldi S., Villani M., Perrella G. (2009). Nuclear phosphatidylcholine and sphingomyelin metabolism of thyroid cells changes during stratospheric balloon flight. J. Biomed. Biotechnol..

[64] Iarmarcovai G., Ceppi M., Botta A., Orsière T., Bonassi S. (2008). Micronuclei frequency in peripheral blood lymphocytes of cancer patients: A meta-analysis. Mutat. Res..

[65] Vrndić O.B., Milošević-Djordjević O.M., Mijatović Teodorović L.C., Jeremić M.Z., Stošić I.M., Grujicić D.V., Zivancević Simonović S.T. (2013). Correlation betweenmicronuclei frequency in peripheral blood lymphocytes and retention of 131-I in thyroid cancer patients. Tohoku J. Exp. Med..

[66] Dom G., Tarabichi M., Unger K., Thomas G., Oczko-Wojciechowska M., Bogdanova T., Jarzab B., Dumont J.E., Detours V., Maenhaut C. (2012). A gene expression signature distinguishes normal tissues of sporadic and radiation-induced papillary thyroid carcinomas. Br. J. Cancer.

[67] Port M., Boltze C., Wang Y., Röper B., Meineke V., Abend M. (2007). A radiation-induced gene signature distinguishes post-Chernobyl from sporadic papillary thyroid cancers. Radiat. Res..

[68] Maenhaut C., Detours V., Dom G., Handkiewicz-Junak D., Oczko-Wojciechowska M., Jarzab B. (2011). Gene expression profiles for radiation-induced thyroid cancer. Clin. Oncol..

[69] Kaiser J.C., Meckbach R., Eidemüller M., Selmansberger M., Unger K., Shpak V., Blettner M., Zitzelsberger H., Jacob P. (2016). Integration of a radiation biomarker into modeling of thyroid carcinogenesis and post-Chernobyl risk assessment. Carcinogenesis.

[70] Selmansberger M., Kaiser J.C., Hess J., Güthlin D., Likhtarev I., Shpak V., Tronko M., Brenner A., Abend M., Blettner M. (2015). Dose-dependent expression of CLIP2 in post-Chernobyl papillary thyroid carcinomas. Carcinogenesis.

[71] Selmansberger M., Feuchtinger A., Zurnadzhy L., Michna A., Kaiser J.C., Abend M., Brenner A., Bogdanova T., Walch A., Unger K. (2015). CLIP2 as radiation biomarker in papillary thyroid carcinoma. Oncogene.

[72] Das S.C., Isichei U.P. (1989). Serum and thyroid tissue lipids in patients with thyroid tumors in euthyroidism. Indian J. Exp. Biol..

[73] Raffelt K., Moka D., Süllentrop F., Dietlein M., Hahn J., Schicha H. (2000). Systemic alterations in phospholipid concentrations of blood plasma in patients with thyroid carcinoma: An in-vitro ^31^P high-resolution NMR study. NMR Biomed..

[74] Guo S., Qiu L., Wang Y., Qin X., Liu H., He M., Zhang Y., Li Z., Chen X. (2014). Tissue imaging and serum lipidomic profiling for screening potential biomarkers of thyroid tumors by matrix-assisted laser desorption/ionization-Fourier transform ion cyclotron resonance mass spectrometry. Anal. Bioanal. Chem..

[75] Rath G., Schneider C., Langlois B., Sartelet H., Morjani H., Btaouri H.E., Dedieu S., Martiny L. (2009). De novo ceramide synthesis is responsible for the anti-tumor properties of camptothecin and doxorubicin in follicular thyroid carcinoma. Int. J. Biochem. Cell Biol..

[76] Törnquist K. (2013). Sphingosine 1-phosphate and cancer: Lessons from thyroid cancer cells. Biomolecules.

